# Functional Aerophagia in a Pediatric Patient with Abdominal Distension

**DOI:** 10.4274/balkanmedj.galenos.2019.2019.6.66

**Published:** 2019-10-28

**Authors:** İrem İnanç, Ümit Nusret Başaran

**Affiliations:** 1 Department of Pediatric Surgery, Trakya University School of Medicine, Edirne, Turkey

A 4-year-old girl was brought to our pediatric emergency department with abdominal distention. She had no complaints of constipation, vomiting, loss of appetite, or abdominal pain. Physical examination revealed an immensely distended abdomen with normoactive bowel sounds. No tenderness, guarding, muscular rigidity, hepatosplenomegaly, or mass was noted on palpation. Percussion detected tympanic sounds in the entire abdomen. Spontaneous gas discharge occurred during rectal examination. No additional pathology or signs of systemic infection were identified, and laboratory parameters were normal. No pathological markers were found in the stool specimen. Weight and height measurements were within the 25^th^ and 50^th^ percentiles, respectively. Plain abdominal X-ray revealed esophageal air sign, large stomach, and generalized gas image in the intestines (Figure 1). However, no evidence of bowel obstruction was observed. Approximately 300 cc of air was evacuated using a nasogastric catheter. Consequently, abdominal distension significantly decreased. Further investigation of the patient’s anamnesis revealed that her complaints started about 3 months ago when her brother was born, indicative of emotional stress factor. No abdominal distension was noted when she woke up in the morning; instead it had worsened throughout the evening, and her swallowing sounds had changed. According to her parents, she burped heavily during the day, passed gas in her sleep, and her room had a malodor. Eventually, the patient was diagnosed with functional aerophagia and referred to the pediatric psychiatry outpatient clinic. Written informed consent was obtained from the patient’s father.

Aerophagia is a functional gastrointestinal disorder characterized by abdominal distention, belching, and gas accumulation because of repeated air swallowing. The condition is known as pathologic aerophagia when accompanied by gastrointestinal symptoms, such as abdominal pain and decreased appetite (1), and it can lead to gastrointestinal perforation. According to the Rome IV criteria, this disorder is included in the classification of childhood and adolescent functional gastrointestinal disorders. Notably, the symptoms should be present for at least 2 months and cannot be explained by another medical condition (2). The early diagnosis of aerophagia is crucial to avoid unnecessary diagnostic tests and possible complications. A plain X-ray is an effective diagnostic tool for this disorder.

Esophageal air sign is a result of excessive air swallowing and may occur when the cricopharyngeal sphincter does not properly close. However, it may not be visible on the radiograph of a normal completely inflated chest (3). Notably, aerophagia reportedly occurs often in adults and children with intellectual disabilities. It has been associated with emotional stress in otherwise healthy children, like our patient (1). Literature review revealed a case of colon perforation from aerophagia, wherein nasogastric decompression was applied (4); however, no standard treatment has been defined because few cases have been reported. Nonetheless, patients should be aware of swallowing air and avoiding excessive intake of carbonated beverages. Furthermore, behavioral therapies (1), with or without medications, such as clonazepam, can be used to reduce psychological stress (5).

## Figures and Tables

**Figure 1:**
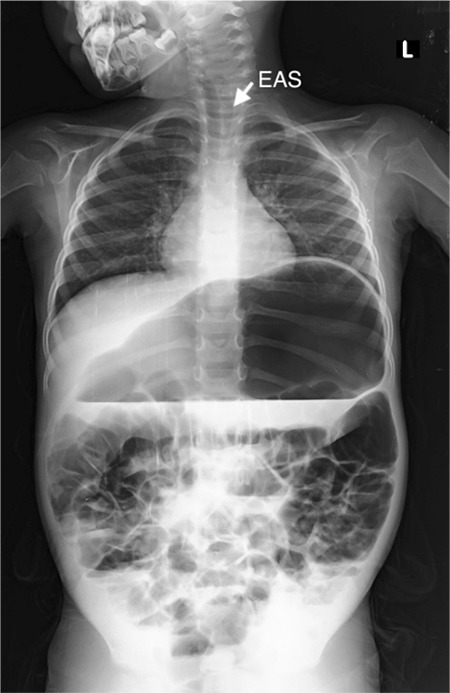
Plain abdominal X-ray shows esophageal air sign and distended stomach, indicative of aerophagia. EAS: esophageal air sign
